# Argentum-Nitricum—Mental and Nervous Symptoms

**Published:** 1890-01

**Authors:** W. M. Butler

**Affiliations:** Brooklyn, N. Y.


### ARGENTUM NITRICUM—MENTAL AND NERVOUS SYMPTOMS.

W. M. Butler, M. D., Brooklyn, N. Y.

Few remedies by their provings have shown greater affinity for the nervous system than Argentum-nitricum. Brain and spinal cord alike in a large number of provers evinced marked evidence of functional disturbance of a nature to produce serious organic lesions. To the consideration of some of these symptoms and their clinical significance we now invite your attention.

The mental symptoms developed, although not as numerous and varied as under many other drugs, are characteristic and important. Certain provers while under its influence seemed almost entirely devoid of brain power. Imbecile in appearance, their weakened memories, childish talk, and inability to fix their minds upon any subject, makes a vivid picture of dementia. In other provers various delusions of a depressing character appear, these, with their hypochondriacal anxiety about their sufferings, and belief that they are neglected and despised by their families, with fear of death and a belief that all their business schemes will fail, and their souls ultimately be lost, remind us of melancholia. Thoughts of suicide, especially by drowning, also occur. A peculiar mental anxiety is also developed, which forces the patient to be constantly busy, still he accomplishes nothing. Constantly in a hurry he hastens to fulfill every engagement, certain that he will be too late, although he may have an hour to spare.

A marked peculiarity of the drug is its development of illusions of sight, as, for instance, when walking the streets the corners of houses seem to project so that the person fears that he will run against them. The sight of high houses causes dizziness, and the impression that the houses on both sides would approach and crush him. Another characteristic hallucination is that he sees snakes around him, upon himself and all the objects about him. Upon the strength of this hallucination we once cured a severe case of melancholia with Argent-nit.[cc] after numerous other drugs had utterly failed. Another symptom similar to that of formication, common in many nervous diseases, is that of a creeping, crawling, itching sensation upon the scalp, as of vermin, or as if the roots of the hair were pulled, causing a constant desire to scratch.

While not as frequently demanded in mental as general nervous disease, when indicated Argentum-nit. will prove speedily and permanently efficacious.

Prominent among the head symptoms we find vertigo, appearing under different conditions—Vertigo in the morning, as if she were turning in a circle, causing her to sit down to prevent falling; vertigo with complete, though transient, blindness, general debility of the limbs and trembling; vertigo and staggering gait; vertigo when walking with eyes closed. Staggers when walking in the dark; has to seize hold of things. On stooping while walking, he staggers. These symptoms of dizziness with defective co-ordination have been repeatedly verified, and have proven most valuable helps in the assignment of this drug to its most important place in therapeutics. Farrington asserts that vertigo is almost always present when Argent-nit. is the remedy.

Several important headaches also belong to this drug.

Among the provings we find excessive congestion of the head, with heaviness and stupefying dullness of the head. Pain in the head, the head appearing enlarged, if on only one side of the head the eye of the affected side appears enlarged. Boring and digging in the left frontal eminence. Digging and tumultuous raging in right hemisphere of brain until he lost his senses. Digging, cutting motion through left hemisphere of the brain, extending from the occiput to the frontal protuberance. Infraorbital neuralgia left side. Neuralgia of head and face, which always takes away her eyesight. In regard to these pains Farrington says: "It is one of the best remedies we have for hemicrania. This is not a simple neuralgia. It is a deep-seated neurotic disease, and by some is supposed to be of epileptic nature. It comes periodically; for its relief the remedy under consideration is one of the best. There is frequently boring pain in the head, which is worse in the left frontal eminence.

This boring is relieved by tight bandaging of the head, hence the wearing of a tight-fitting silk hat relieves. It is excited by any mental emotion of an unpleasant kind, or by anything that depreciates the nervous system, as loss of fluids, loss of sleep or mental strain. Sometimes, the pains become so severe that the patient loses his senses. The paroxysms frequently culminate in vomiting of bile or sour fluids."

Arg-nit. is also useful in a peculiar intense neuralgia characterized by a sensation as if the bones of the head were separating, or the head was enormously large; also in prosopalgia, when the infraorbital branches of the fifth pair and the nerves supplying the teeth are involved.

In both of these forms of neuralgia the pain may be so severe as to produce a loss of consciousness, and is usually attended by a sour taste in mouth, and terminates with sour or bilious vomiting.

We recently relieved a severe case of facial neuralgia with Argent-nit. guided by the characteristic desire for sugar, the patient being obliged to eat a coffee-cupful at a time to satisfy the longing.

Another neuralgia, in which this remedy is often indicated, is that of the stomach. It is especially adapted to nervous women, when the pain has been produced by excitement and loss of sleep, or occurs during the menses. The patient complains of a lump in the stomach, and a gnawing, burning, griping pain, which, commencing in the pit of the stomach, radiates in every direction. This is accompanied by a feeling of great distension as if the stomach would burst. The pain is aggravated by the slightest amount of food, and relieved by bending double and firm pressure. These paroxysms end by the vomiting of stringy, gluey, tenacious mucus and the eructation of enormous quantities of wind.

We also find among the recorded symptoms severe pains through the chest, with irregularity or intermittance in the action of the heart, inability to breathe, with cold face and hands and faintish nausea. This vivid picture of angina pectoris suggested its use in this disease, and the clinical results have verified the correctness of the proving.

Farrington accords to this remedy a high rank in the cure of epilepsy, which has been caused by fright, or occurs during menstruation. He gives as the strong indicating symptom in this disease dilated pupils, for days or hours before the attack, the patient being exceedingly restless, with great tremulousness of the hands after the seizure.

In no nervous disease has this drug been more frequently credited with efficiency than in locomotor ataxy. Nor have the provings of any other drug presented more symptoms analogous to those recognized as characteristic of this disease. The intense vertigo in the dark and inability to stand with the eyes closed, general defectiveness in muscular co-ordination, shooting pains in different parts of the body, band-like constriction of bowels, epigastrium, chest, or waist, paralytic symptoms of bladder, numbness of finger-tips, legs, and feet, formication of arms and legs, general tremulousness, with weakness and exhaustion make an almost complete portrait of the disease, and warrant us in expecting results from this drug when applied to these cases. When positive organic changes have taken place in the cord we do not believe that a cure can be effected by any medicine, but in the early stages, before any permanent lesion has occurred, we know, from the overwhelming testimony of numerous writers in our school, and from our own experience, that much good can be accomplished from the exhibition of Argent-nit.

If Argentum-nitricum had proved utterly useless in every field of disease, except that of the nervous system, we should still be under countless obligations to its provers for their services in assigning it a permanent place in our medical armamentarium.

